# Mitogen-stimulated cell proliferation and cytokine production in major depressive disorder patients

**DOI:** 10.1186/s12888-018-1906-5

**Published:** 2018-10-12

**Authors:** Ping Lin, Bingyu Ding, Yunqiang Wu, Ke Dong, Qingtian Li

**Affiliations:** 1 Department of Medical Laboratory, Shanghai Mental Health Center, Shanghai Jiao Tong University School of Medicine, Shanghai, 200030 China; 2 Department of Laboratory Medicine, Ruijin Hospital, Shanghai Jiao Tong University School of Medicine, Shanghai, 200025 China; 3 Department of Immunology and Microbiology, Shanghai Jiao Tong University School of Medicine, Shanghai, 200025 China

**Keywords:** Chronic unpredictable mild stress, Cytokine, Major depressive disorder, Mitogen

## Abstract

**Background:**

Major depressive disorder (MDD) is related to human’s immune status, and immunological indicators such as mitogen stimulated cell proliferation and cytokines may become candidate biomarkers for disease diagnosis.

**Methods:**

One hundred diagnosed major depressive disorder subjects and 100 health controls were enrolled in this study. Phytohaemagglutinin and lipopolysaccharide stimulated cell proliferations and cytokine concentrations were detected in peripheral blood mononuclear cells from both groups. The corresponding stimulated responses were conducted and confirmed in chronic unpredictable mild stress (CUMS) mice.

**Results:**

Compared to the people in control group, there were lower cell proliferations and lower TNF-α produced in lipopolysaccharide stimulated peripheral blood mononuclear cells in depression patients, lower IL-2 and IL-10 produced in phytohaemagglutinin stimulated peripheral blood mononuclear cells in depression patients, higher IL-6, IL-10 and lower IL-2 secretions were detected in peripheral plasma in depression patients. In CUMS mice we found lower splenocyte proliferations, lower IL-1α productions and higher IL-6 secretions in lipopolysaccharide stimulated splenocytes. It seems lipopolysaccharide stimulated cell proliferation activities were inhibited in depressive states.

**Conclusions:**

Lower lipopolysaccharide stimulated cell proliferation and phytohaemagglutinin stimulated or plasma cytokine IL-2 decreases should be potential monitoring indices in the depressive state assessment for major depressive disorder patients.

## Background

Major depressive disorder (MDD) is a mental illness affective disorder, differing levels of symptomatic severity, and leading self-harm and suicide attempts [1]. With the increasing pressure in work and live, the incidence of MDD is keeping increasing every year, and the World Health Organization (WHO) predicted that the incidence will reach to 10% of the total population by 2020 and become the second most common disease of the world.

MDD is a complex disorder with multiple mechanisms. In general consideration, biochemical, neurochemical, genetic, social environmental, even microecological factors are involved in the progression of depression, however, its real cause and pathogenesis remains unclear [2]. There are close links between the outbreak of MDD and immune factors such as cytokines and other immune regulatory molecules. For example, Harison [3] revealed the depression-like behavior is closely related to the changes of cytokines concentration.

To evaluate the immune status in MDD patients, here we analyzed the peripheral blood mononuclear cells (PBMCs) cytokine production and cell proliferation stimulated by mitogen lipopolysaccharide (LPS) and phytohemagglutinin (PHA). At the same time, we created a chronic unpredictable mild stress (CUMS) mouse model to illustrate the corresponding cytokine responses from mouse splenocytes stimulated by mitogen LPS and PHA. Compare to many other alternatives, CUMS method is closer to the natural course of development of depression.

## Methods

### Specimen collection and PBMCs separation, culture and mitogen stimulating

One hundred MDD patients were recruited in this study. They were defined according to the Diagnostic and Statistical Manual of Mental Disorders, fourth edition (DSM-IV) (American Psychiatric Association, 1994) and 17-items Hamilton Depression Rating Scale (HAM-D) scales ≥23. Another 100 healthy people were from the health examination center without any personal or family history of psychiatric disorders. Subjects having current infections, or a present and past history of autoimmune disorders that might influence the inflammatory states were excluded.

We collected peripheral blood samples in 4 ml ethylenediaminetetraacetic acid (EDTA) tubes and transported to the lab at room temperature within 30 min. The plasma was obtained by blood centrifugation at 1500×g for 7 min and kept frozen at − 80 °C. The PBMCs were isolated using density gradient centrifugation through Ficoll-Hypaque. The PBMCs were suspended in RPMI 1640 medium, containing L-glutamine 1% and antibiotics (penicillin 100 U/ml–streptomycin 100 μg/ml) with 10% heat-inactivated fetal calf serum, seeded in 12 well cell culture cluster and cultured with 100 ng/ml LPS or 10 μg/ml PHA respectively at 37 °C in a humidified 5% CO_2_ cell culture incubator. Cells and supernatants were collected separately 48 h later, aliquoted, and stored at − 80 °C before use.

### MTT colorimetric assay

MTT cell proliferation and cytotoxicity assay kit (Beyotime Biotechnology, Shanghai, China) was used in this study for the cell proliferation assay of PBMCs stimulated by LPS and PHA. The human PBMCs were resuspended to a concentration of 2 × 10^4^ cells/mL. A 100 μL aliquot containing 2 × 10^3^ cells was added immediately to each well of a 96-well flat bottom microtitre plate in triplicate. LPS or PHA were added to the wells to the final concentration of 100 ng/ml (LPS) and 10 μg/ml (PHA). After a period of 72 h incubation at 37 °C in 5% CO_2_, PBMCs proliferation was assayed with MTT kit.

### Elisa

The PBMCs cytokine (IL-1α, IL-2, IL-6, IL-10, TNF-α, and IFN-γ) concentrations were determined by a sandwich enzyme-linked-immunosorbent assay using a commercially available kit (Beijing 4A Biotech Co., Ltd. China).

### Chronic unpredictable mild stress (CUMS) model and its verification

Twenty male Balb/c mice from Shanghai Laboratory Animal Co. Ltd.(Shanghai, China) were used to verify the cytokine production of MDD patients in this study. All animals were comfort feeding at 19–21 °C except the special requirement in CUMS group. After 1 week of acclimatization, equal amount of mice were randomly sorted into CUMS and control groups respectively. Next, CUMS mice were subjected to various and repeated unpredictable mild stressors for a period of 4 weeks [4]. Mice in the control group were also single housed and were provided with standard daily care.

Tail suspension test and forced swim test were used to confirm the depression status of CUMS mice [5]. Immobility time was detected in these two tests and touch times and swim distance were measured in forced swim test.

### Mitogen stimulated splenocyte proliferation and cytokine production in CUMS model

On the day after the tail suspension test and forced swim test, all the mice in CUMS group and control group were euthanized by CO_2_ inhalation or cervical dislocation, and their splenocytes and peripheral plasma were collected for mitogen stimulated splenocyte proliferation and cytokine production tests. The procedures of cell culture, stimulating, and cytokines measure methods were the same as those used in human specimens except using mouse cytokine ELISA kits (Beijing 4A Biotech Co., Ltd. China). All subjects were measured three times on the same day.

### Statistical analysis

Demographic and clinical variables of the patient and healthy control groups were analyzed by t-test for continuous variables and chi-squared for discrete variables. The statistical software SPSS17.0 was used in this study. Data were presented as means ± SD. Differences at *p* < 0.05 were considered significant.

## Results

### Demographic data

The demographic data of MDD patients and control subjects in this study were showed in Table 1. There was no difference in gender, age, BMI and education years between these two groups.

**Table 1:** Demographics of depressed patients and control subjects

parameter	MDD (*n* = 100)	Control (*n* = 100)	*P* value
Gender (F/M)	58/42	62/38	0.66
Age (years; means±SD)	36.5 ± 6.5	35.3 ± 5.9	0.17
BMI (means±SD)	21.8 ± 4.7	22.6 ± 3.9	0.19
Education years (means±SD)	13.3 ± 4.8	14.4 ± 3.6	0.07
Smoking/No smoking	40/60	32/68	0.30
Duration of illness (years)	4.6 ± 1.8		

### MTT colorimetric assay and cytokine levels in mitogen stimulated PBMCs systems and peripheral plasma

**Fig. 1:**
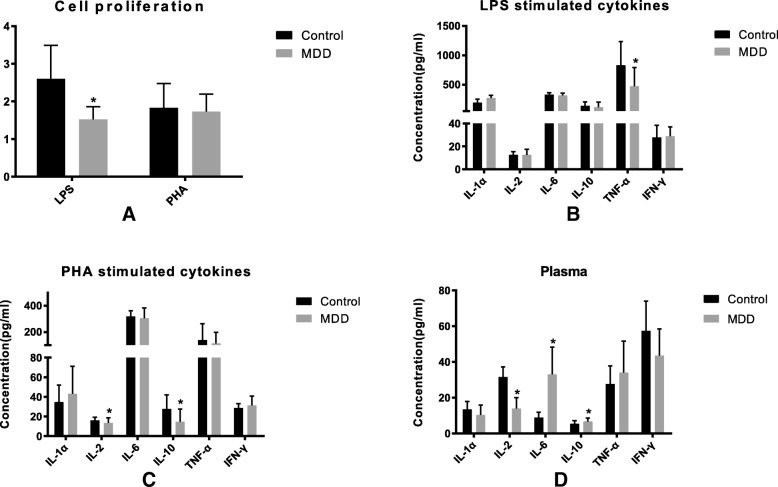
PBMCs from 100 MDD patients and another 100 healthy people were stimulated by LPS and PHA, then the cell proliferation levels were detected with a MTT kit and cytokine IL-1α, IL-2, IL-6, IL-10, TNF-α and IFN-γ were detected with ELISA kits. **a** The LPS and PHA stimulated PBMCs proliferation ratio (test/control) in MDD patients. **b** Cytokines of PBMCs after LPS stimulation; **c** Cytokines of PBMCs after PHA stimulation; **d** Cytokines in plasma. Data presented as means ± SD. Significantly different from controls were signed with * (*p* < 0.05)

MTT test was used to detect the stimulating effects on human PBMCs by LPS and PHA. The proliferation ratio (test / control) analyzed at 570 nm with ELISA reader are shown in Fig. 1. The LPS stimulating effects on PBMCs proliferation in MDD group were significantly lower than those in control group. For the PHA stimulating assay, there was no significant differences between the proliferation rate in the two groups.

Double antibody sandwich ELISA kits were used in this study to evaluate various cytokine concentrations in each group. As shown in Fig. 1, when the PBMCs were stimulated by LPS, there was lower TNF-α in MDD group than those in control group, while no difference in IL-1α, IL-2, IL-6, IL-10, and IFN-γ between the two groups. In PHA stimulated branch, lower IL-2 and IL-10, rather than IL-1α, IL-6, TNF-α, and IFN-γ were detected in MDD group than those in control group. For the cytokine production in peripheral plasma, more IL-6 and IL-10 and less IL-2 were found in MDD group than control group.

### Behavioral verification and mitogen stimulated splenocyte proliferation and cytokine production in CUMS model

**Fig. 2:**
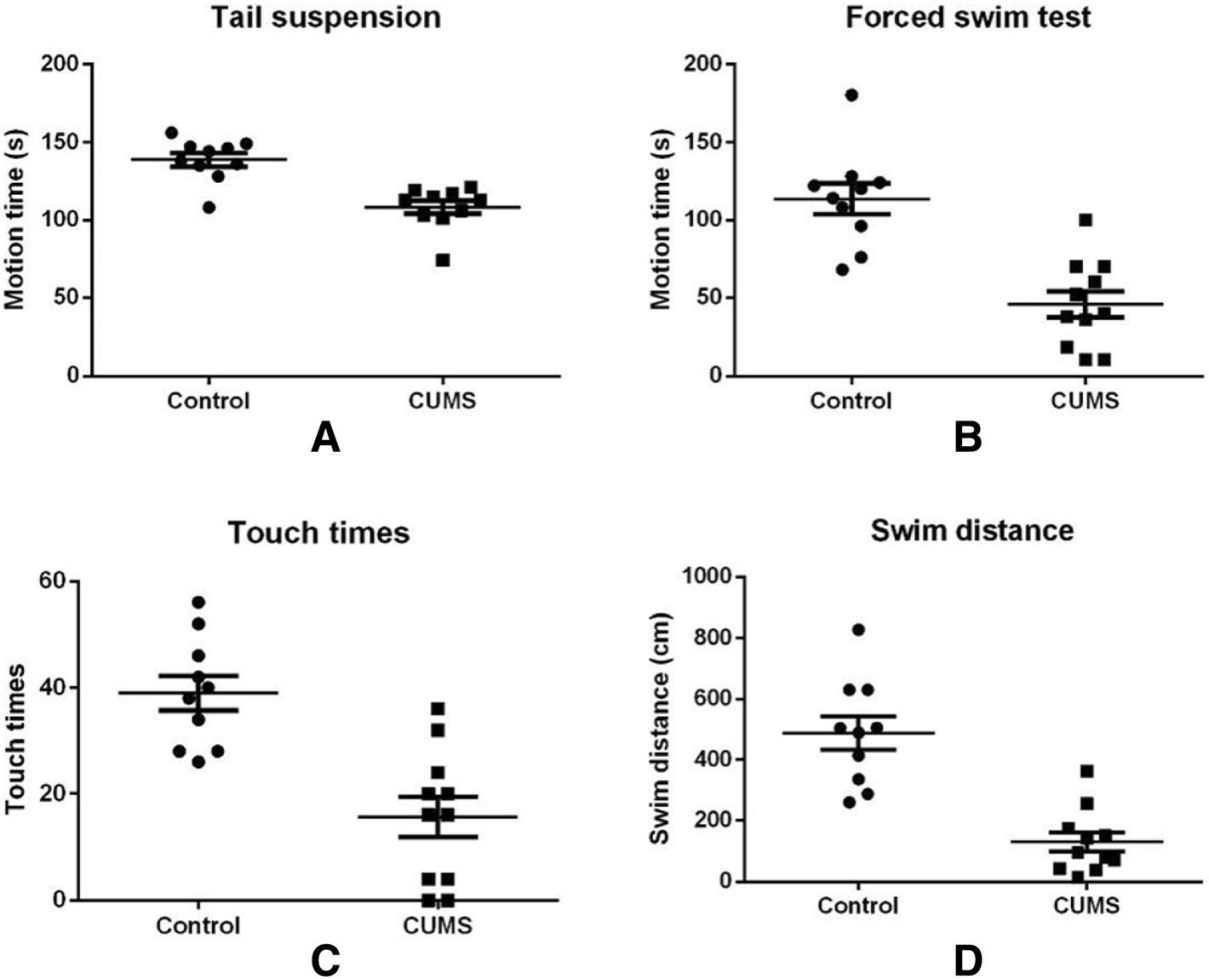
Ten Balb/c mice were treated with a chronic unpredictable mild stress (CUMS) and another 10 mice were feed routinely as control. Mice in CUMS group showed lower activities in behavioral verification as **a** tail suspension; **b** motion time, **c** touch times, and **d** swim distances in forced swim test. Data presented as means ± SD. Significantly different from controls were signed with * (*p* < 0.05)

Motion times were detected in tail suspension test and forced swim test, touching wall times and swimming distances were used to evaluate MDD model in forced swim test too. As shown in Fig. 2, motion time and swimming time, touching wall times and swim distances were significantly decreased in CUMS mice than those in control group.

**Fig. 3:**
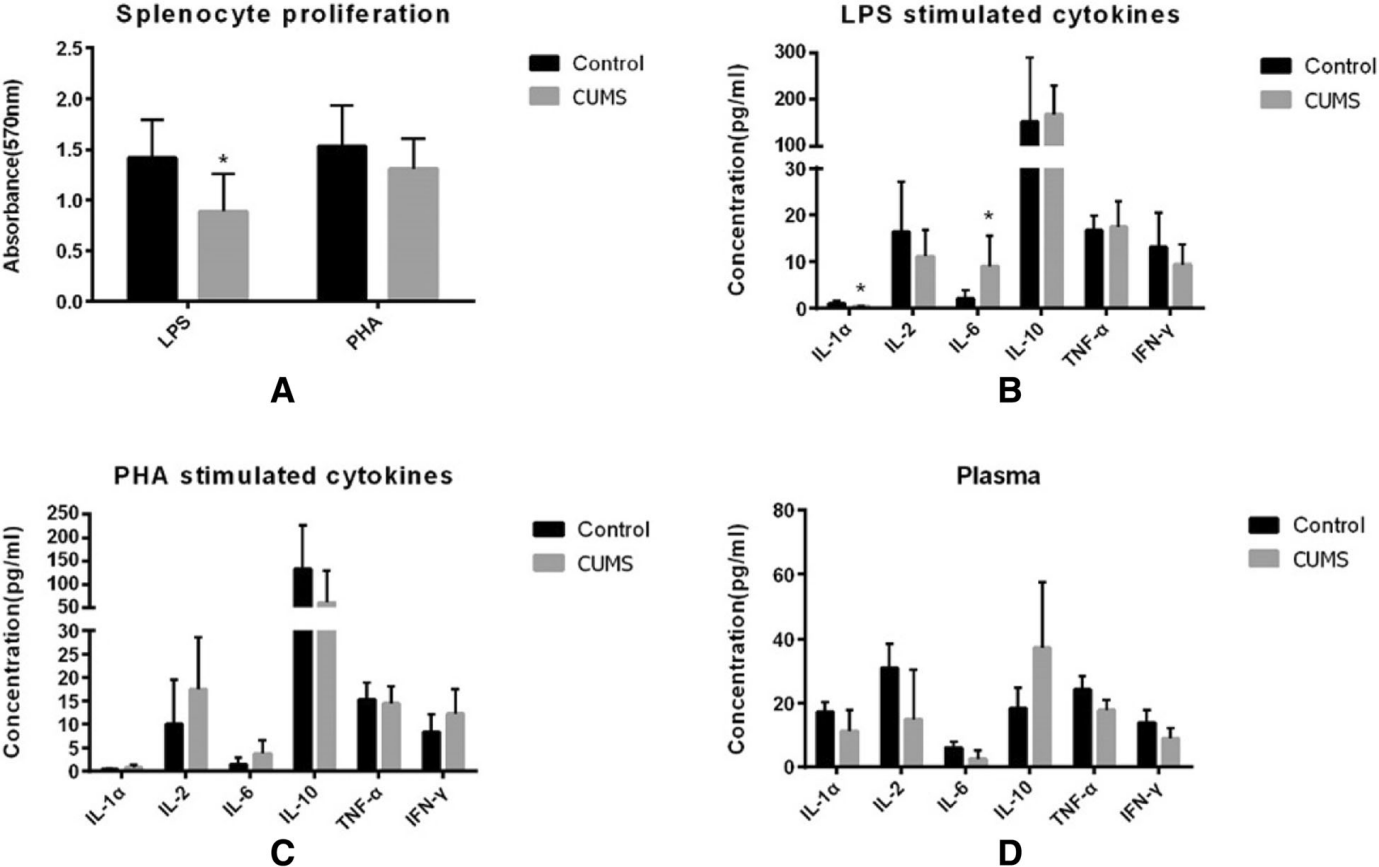
Ten Balb/c mice were treated with a chronic unpredictable mild stress (CUMS) and another 10 mice were feed routinely as control. **a** Splenocyte proliferation reaction, cytokine responses under **b** LPS and **c** PHA stimulating, and **d** plasma cytokine concentration were detected in CUMS and control groups. Data presented as means ± SD. Significantly different from controls were signed with * (*p* < 0.05)

In splenocyte proliferation and cytokine production in CUMS tests, we found a lower splenocyte proliferation in LPS stimulated CUMS mice. In mitogen stimulated cytokine detections, there were lower IL-1α and higher IL-6 in LPS stimulated splenocytes in CUMS mice as compared with control group (Fig. 3). There was no difference in cytokines production of peripheral plasma and splenocyte stimulated by PHA between the two groups.

## Discussion

The immune system plays an important role in the development of depression. There is a lot of evidence that immune dysfunction is involved in the pathogenesis of MDD [6]. The relevant conclusions came from two fields: the relationship between inflammatory signaling and cytokines in the pathogenesis of MDD, and the improvement of the therapeutic effect and prognosis with the adjunctive treatment using anti-inflammatory drugs to improve the therapeutic effect [7, 8]. Among the numerous immune related indicators, it showed that cytokine-mediated immune dysfunction and pro-inflammatory cytokines have an effect on the pathogenesis of MDD [9].

In this study, PBMCs proliferation assays along with LPS and PHA were used to describe the immune responses to mitogen LPS and PHA. PHA and LPS are the most classical mitogens targeting T and B cells respectively. They have no antigen specificity for cell activation, and mainly reflecting the overall immune response of cells and individuals. In LPS stimulation branch, the MDD group showed a lower proliferation compared to the control group, while there was no difference in the PHA effects between MDD group and the control group in the PHA branch. Mitogen LPS and PHA stimulating assay can discover the whole immune response and proliferation levels of the B and T lymphocyte. These indicators can also help to understand the courses and outcomes of psychiatric disorders including MDD [10]. Here we found the B cell related immune proliferations were impaired in MDD patients. B cell counts was found to be diminished in Pavon’s study [11], it seems that B cell proliferation may be diminished with the interactions between neuroendocrine and immune systems in MDD patients. Here the LPS-stimulating PBMCs showed lower TNF-α in MDD group than those in control group. This lower TNF-α was found in a depressive status of systemic lupus erythematosus [12]. As a major component of environmental and pathogenic microbial products, LPS can not only drive differential T cell polarization but also activate B cells and other antigen presenting cells [13]. A low LPS response level may be understood as a change on body’s immune response ability against bacterial infections or other bacterial related chronic inflammation [14]. We showed that decreased PHA-stimulated IL-2 and IL-10 production in MDD group than control group. IL-10 is a cytokine secreted by Th2-type cells, which suppressed Thl-type cytokines’ mRNA transcription, inhibited the cellular immune response. We also found that decreased LPS-stimulated TNF-α production in MDD group compared to control group.

Th1 and Th2 immune cells maintain human’s normal immune function exist in the human body with its relatively balanced ratio and maintain the body’s normal immune function. This balance may take on a unique role in the development of MDD and other psychiatric disorders [15]. In the present study, we detected the expression of classical Thl type cytokines by IL-2, IFN-γ and TNF-α, the expression of classical Th2 type cytokines by IL-10 [16]. We found a great degree of cytokine reductions in Th2 cells than those in Th1 cells. It demonstrated that Thl response in MDD patients displayed a dominant state relatively. The different immune states may help differentiated the syndrome of major depression [17]. The ratio of Th1/Th2 balance appeared a shift to Th1 cells in MDD patients. In view of the fact that some antidepressants can move the Th1/Th2 balance to Th2 response [18], it is necessary to study the mechanism of this state of shift, so that we can selective adjustment this balance, which may reverse the drift of the state, so as to achieve the treatment purpose of MDD patients. In addition, TNF-α, INF-γ, IL-2, and IL-6 were defined as inflammatory cytokines, while IL-10 was considered as anti-inflammatory cytokines or autoimmune cytokines. Overall, our cytokine data did not show the classic inflammatory response in MDD patients. Maybe MDD or some other mental illnesses can not be simply regarded as an inflammation similar to bacterial infections.

Here we successfully created a CUMS model and detected the immune factor changes in it. There was a lower splenocyte proliferation in LPS stimulated CUMS mice compared to control group. We found lower IL-1α and higher IL-6 in LPS stimulated splenocytes in CUMS mice compared with control group. As an amyloid inducer, higher IL-1α can improve the amyloid production. This result might indicate that a relatively short duration of adverse stimuli is not sufficient to a fully mimic chronic onset of MDD [19]. We didn’t find any difference in PHA stimulated splenocytes and peripheral plasma between CUMS mice and control mice.

Most of psychiatric disorders are related to human immune system and the regulations and interactions between those cytokines and immune molecules [20]. In fact, there are extensive interactions between different immune molecules and neurotrophic factors. Müller et al. have found pro-inflammatory cytokines activate indoleamine 2,3-dioxygenase (IDO) [21], which is an important component in regulate the metabolism of tryptophan (TRP) [22]. Owing to the catabolism of TRP, it induces a halt in the lymphocyte cell cycle and control the proliferation of lymphocyte. Our research is more focused on MDD patients with a chronic course of illness, which may support Müller’s conclusion in aspect of immune state. Here we detected a series of changed factors in LPS stimulated PBMCs, PHA stimulated PBMCs, and plasma in MDD patients and in CUMS mice respectively. These changes might be combined with neural factors and become future biomarkers for MDD diagnosis and course monitoring.

In fact, MDD is not only a severe psychiatric syndrome itself, but also an important hazard that affects the diagnosis, treatment and progression of other diseases [23]. MDD is not an simple inflammation similar to bacterial infections, its parameters should be a reasonable combination or formula operation of several factors. In addition to scale assessment, imaging features, blood transcriptome and proteome [24], brain-gut-axis and intestinal microflora [2], together with immune and nerve-humoral parameters should be ideal candidates in the coming combination or pattern [25].

## Conclusions

Lower LPS stimulated cell proliferation were confirmed in MDD patients PBMCs and CUMS mice splenocytes. Further studies should focus on how to use the immune disorder to evaluate and supervise the efficacy and disease outcomes in MDD patients.

## Abbreviations

CUMS: Chronic unpredictable mild stress
DSM-IV: Diagnostic and Statistical Manual of Mental Disorders, fourth edition
EDTA: Ethylenediaminetetraacetic acid
HAM-D: Hamilton depression rating scale
IDO: Indoleamine 2, 3-dioxygenase
IRB: Institutional review board
LPS: Lipopolysaccharide
MDD: Major depressive disorder
MTT: 3-(4, 5-dimethylthiazolyl-2)-2, 5-diphenyltetrazolium bromide
PBMCs: Peripheral blood mononuclear cells
PHA: Phytohemagglutinin
TRP: Tryptophan
WHO: World Health Organization

## References

[1] Kessler RC, Berglund P, Demler O, Jin R, Koretz D, Merikangas KR, Rush AJ, Walters EE, Wang PS (2003). The epidemiology of major depressive disorder: results from the National Comorbidity Survey Replication (NCS-R). Jama.

[2] Lin P, Ding B, Feng C, Yin S, Zhang T, Qi X, Lv H, Guo X, Dong K, Zhu Y (2017). Prevotella and Klebsiella proportions in fecal microbial communities are potential characteristic parameters for patients with major depressive disorder. J Affect Disord.

[3] Harrison NA, Brydon L, Walker C, Gray MA, Steptoe A, Dolan RJ, Critchley HD (2009). Neural origins of human sickness in interoceptive responses to inflammation. Biol Psychiatry.

[4] Bai YY, Ruan CS, Yang CR, Li JY, Kang ZL, Zhou L, Liu D, Zeng YQ, Wang TH, Tian CF (2016). ProBDNF Signalling regulates depression-like Behaviours in rodents under chronic stress. Neuropsychopharmacology.

[5] Pesarico AP, Sartori G, Bruning CA, Mantovani AC, Duarte T, Zeni G, Nogueira CW (2016). A novel isoquinoline compound abolishes chronic unpredictable mild stress-induced depressive-like behavior in mice. Behav Brain Res.

[6] Miller AH (2010). Depression and immunity: a role for T cells?. Brain Behav Immun.

[7] Kopschina Feltes P, Doorduin J, Klein HC, Juarez-Orozco LE, Dierckx RA, Moriguchi-Jeckel CM, de Vries EF (2017). Anti-inflammatory treatment for major depressive disorder: implications for patients with an elevated immune profile and non-responders to standard antidepressant therapy. J Psychopharmacol.

[8] Leonard B, Maes M (2012). Mechanistic explanations how cell-mediated immune activation, inflammation and oxidative and nitrosative stress pathways and their sequels and concomitants play a role in the pathophysiology of unipolar depression. Neurosci Biobehav Rev.

[9] Davami MH, Baharlou R, Ahmadi Vasmehjani A, Ghanizadeh A, Keshtkar M, Dezhkam I, Atashzar MR (2016). Elevated IL-17 and TGF-beta serum levels: a positive correlation between T-helper 17 cell-related pro-inflammatory responses with major depressive disorder. Basic Clin Neurosci.

[10] Rapaport MH, Bresee C (2010). Serial mitogen-stimulated cytokine production from continuously ill patients with schizophrenia. Neuropsychopharmacology.

[11] Pavon L, Sandoval-Lopez G, Eugenia Hernandez M, Loria F, Estrada I, Perez M, Moreno J, Avila U, Leff P, Anton B (2006). Th2 cytokine response in major depressive disorder patients before treatment. J Neuroimmunol.

[12] Postal M, Lapa AT, Sinicato NA, de Oliveira Pelicari K, Peres FA, Costallat LT, Fernandes PT, Marini R, Appenzeller S (2016). Depressive symptoms are associated with tumor necrosis factor alpha in systemic lupus erythematosus. J Neuroinflammation.

[13] Xu H, Liew LN, Kuo IC, Huang CH, Goh DL, Chua KY (2008). The modulatory effects of lipopolysaccharide-stimulated B cells on differential T-cell polarization. Immunology.

[14] Mogensen TH (2009). Pathogen recognition and inflammatory signaling in innate immune defenses. Clin Microbiol Rev.

[15] Song C, Halbreich U, Han C, Leonard BE, Luo H (2009). Imbalance between pro- and anti-inflammatory cytokines, and between Th1 and Th2 cytokines in depressed patients: the effect of electroacupuncture or fluoxetine treatment. Pharmacopsychiatry.

[16] Li Q, Zhou Y, Dong K, Guo X (2010). Potential therapeutic efficacy of a bactericidal-immunomodulatory fusion peptide against methicillin-resistant Staphylococcus aureus skin infection. Appl Microbiol Biotechnol.

[17] Muller N, Schwarz MJ (2006). Neuroimmune-endocrine crosstalk in schizophrenia and mood disorders. Expert Rev Neurother.

[18] Martino M, Rocchi G, Escelsior A, Fornaro M (2012). Immunomodulation mechanism of antidepressants: interactions between serotonin/norepinephrine balance and Th1/Th2 balance. Curr Neuropharmacol.

[19] Lee KS, Chung JH, Lee KH, Shin MJ, Oh BH, Lee SH, Hong CH (2009). Simultaneous measurement of 23 plasma cytokines in late-life depression. Neurol Sci.

[20] Segerstrom SC, Miller GE (2004). Psychological stress and the human immune system: a meta-analytic study of 30 years of inquiry. Psychol Bull.

[21] Muller N, Schwarz MJ (2007). The immune-mediated alteration of serotonin and glutamate: towards an integrated view of depression. Mol Psychiatry.

[22] Mellor AL, Munn DH (1999). Tryptophan catabolism and T-cell tolerance: immunosuppression by starvation?. Immunol Today.

[23] Di Stasio D, Candotto V, Serpico R, Migliozzi R, Petruzzi M, Tammaro M, Maio C, Gritti P, Lauritano D, Lucchese A (2018). Depression and distress in burning mouth syndrome: a case control study. J Biol Regul Homeost Agents.

[24] Hori H, Sasayama D, Teraishi T, Yamamoto N, Nakamura S, Ota M, Hattori K, Kim Y, Higuchi T, Kunugi H (2016). Blood-based gene expression signatures of medication-free outpatients with major depressive disorder: integrative genome-wide and candidate gene analyses. Sci Rep.

[25] Mastrangelo F, Frydas I, Ronconi G, Kritas SK, Tettamanti L, Caraffa A, D Ovidio C, Younes A, Gallenga CE, Conti P (2018). Low-grade chronic inflammation mediated by mast cells in fibromyalgia: role of IL-37. J Biol Regul Homeost Agents.

